# HO-1 Modulates Aerobic Glycolysis through LDH in Prostate Cancer Cells

**DOI:** 10.3390/antiox10060966

**Published:** 2021-06-16

**Authors:** Florencia Cascardo, Nicolás Anselmino, Alejandra Páez, Estefanía Labanca, Pablo Sanchis, Valeria Antico-Arciuch, Nora Navone, Geraldine Gueron, Elba Vázquez, Javier Cotignola

**Affiliations:** 1 Laboratorio de Inflamación y Cáncer, Departamento de Química Biológica, Facultad de Ciencias Exactas y Naturales, Universidad de Buenos Aires, Buenos Aires C1428EGA, Argentina; fcascardo@qb.fcen.uba.ar (F.C.); pablosanchis@qb.fcen.uba.ar (P.S.); vantico@qb.fcen.uba.ar (V.A.-A.); ggueron@iquibicen.fcen.uba.ar (G.G.); 2 CONICET-Universidad de Buenos Aires, Instituto de Química Biológica de la Facultad de Ciencias Exactas y Naturales (IQUIBICEN), Buenos Aires C1428EGA, Argentina; 3 Department of Genitourinary Medical Oncology, The David H. Koch Center for Applied Research of Genitourinary Cancers, The University of Texas MD Anderson Cancer Center, Houston, TX 77030, USA; nanselmino@mdanderson.org (N.A.); elabanca@mdanderson.org (E.L.); nnavone@mdanderson.org (N.N.); 4 Unidad de Transferencia Genética, Instituto de Oncología “Dr. Angel H. Roffo”, Facultad de Medicina, Universidad de Buenos Aires, Buenos Aires C1417DTB, Argentina; apaez@qb.fcen.uba.ar

**Keywords:** *HMOX1*, HO-1, LDH, cancer metabolism, prostate cancer

## Abstract

Prostate cancer (PCa) is the second most diagnosed malignancy and the fifth leading cause of cancer associated death in men worldwide. Dysregulation of cellular energetics has become a hallmark of cancer, evidenced by numerous connections between signaling pathways that include oncoproteins and key metabolic enzymes. We previously showed that heme oxygenase 1 (HO-1), a cellular homeostatic regulator counteracting oxidative and inflammatory damage, exhibits anti-tumoral activity in PCa cells, inhibiting cell proliferation, migration, tumor growth and angiogenesis. The aim of this study was to assess the role of HO-1 on the metabolic signature of PCa. After HO-1 pharmacological induction with hemin, PC3 and C4-2B cells exhibited a significantly impaired cellular metabolic rate, reflected by glucose uptake, ATP production, lactate dehydrogenase (LDH) activity and extracellular lactate levels. Further, we undertook a bioinformatics approach to assess the clinical significance of *LDHA*, *LDHB* and *HMOX1* in PCa, identifying that high *LDHA* or low *LDHB* expression was associated with reduced relapse free survival (RFS). Interestingly, the shortest RFS was observed for PCa patients with low *HMOX1* and high *LDHA,* while an improved prognosis was observed for those with high *HMOX1* and *LDHB*. Thus, HO-1 induction causes a shift in the cellular metabolic profile of PCa, leading to a less aggressive phenotype of the disease.

## 1. Introduction

Prostate cancer (PCa) affects men worldwide, being the second most commonly diagnosed malignancy and the fifth leading cause of cancer-related deaths among men [1]. It is a heterogeneous disease with a highly variable prognosis, since many patients present an indolent clinical course and others have an aggressive lethal disease [2].

From the onset of the tumor, and throughout its development, cancer cells acquire different biological capabilities that enable tumor progression [3]. Energetic metabolism is different in cancer cells compared with normal cells due to the increased energy-rich metabolites needed to fuel tumor growth and division [4]. As first described by Otto Warburg, even in the presence of oxygen, most tumors exhibit a high glycolytic rate, producing large amounts of lactate from glucose [5,6]. Despite being less efficient in terms of energy generation, this process, known as aerobic glycolysis or the Warburg effect, seems advantageous, since it promotes the production of several intermediates for the macromolecule synthesis, such as carbohydrates, proteins, lipids and nucleic acids, as well as reducing power in the form of NADPH [7].

In aerobic glycolysis, the interconversion between pyruvate and lactate is catalyzed by the lactate dehydrogenase (LDH) enzyme, of which the most predominant isoforms are LDHA, with higher affinity for pyruvate, and LDHB, with higher affinity for lactate [8,9]. As a consequence, LDHA preferentially converts pyruvate to lactate and NADH to NAD^+^, whereas LDHB favors the reverse reaction [10]. LDHA is commonly overexpressed in cancer cells, leading to an excessive accumulation of lactate and promoting its secretion by the monocarboxylate transporters (MCTs) which, in turn, increases the acidification of the tumor microenvironment [11]. In addition, this acidosis is associated with tumor progression, as it favors angiogenesis, invasion, metastasis, immunosuppression and therapy resistance, highlighting the role of lactate as a key oncometabolite [10].

Peripheral healthy prostatic epithelium has a highly specialized metabolism based on the accumulation of zinc [12,13], which inhibits the mitochondrial enzyme aconitase 2 (ACO2) and enables a physiological truncation of the tricarboxylic acid (TCA) cycle, resulting in an increased secretion of citrate [14,15]. Accordingly, as the NADH reducing equivalents needed to power ATP generation through the oxidative phosphorylation (OXPHOS) are not produced in the TCA cycle, healthy prostate tissue must rely on glycolysis to obtain energy [16,17]. Consistent with the concept that tumors adapt the normal metabolic program of their tissue-of-origin to support their own inappropriate cell proliferation [18], primary prostate tumors are distinguished for presenting unique metabolic features. In PCa, zinc levels are reduced as a consequence of the loss of zinc transporters during oncogenic transformation [13,19], removing ACO2 inhibition and restoring TCA cycle activity [15,17]. Thus, prostatic tumors exhibit enhanced OXPHOS and lipogenesis to support the high proliferative requirements of undifferentiated cells [15,16,20].

Heme oxygenase 1 (HO-1), encoded by the *HMOX1* gene, is the rate-limiting enzyme in heme degradation [21,22] and participation in oxidative stress response and cellular homeostasis is well described [23]. HO-1 has been reported to play an important role in the modulation of cellular bioenergetics [24,25]. Although HO-1 implications in cancer have been studied for the past years, its role is still controversial and seems to depend on the tumor type [26,27,28]. In this context, we have previously shown that HO-1 upregulation is associated with a less aggressive phenotype in PCa, inhibiting cell proliferation, migration and invasion in vitro [29], impairing tumor growth and angiogenesis in vivo and downregulating the expression of genes associated with inflammation, such as the TNF-α axis [29,30]. Additionally, HO-1 interacts with STAT3, interfering with its signaling pathway and, consequently, down-modulating AR transcriptional activity [31]. We have also demonstrated that HO-1 regulates cell morphology through changes in the actin cytoskeleton [32], and increases the expression and membrane localization of E-cadherin and β-catenin in PCa cell lines, favoring a more epithelial phenotype [33]. Moreover, we have shown that pre-treating the stroma of fully immunocompetent mice with hemin, a well-known inducer of HO-1, prior to TRAMP-C1 tumor challenge, translates to a significant increase in tumor latency and a significant decrease in the initial tumor growth rate [34]. Furthermore, we have reported that HO-1 modulates bone remodeling and alters the communication between PCa and bone cells [35]. We have also demonstrated that HO-1 induction affects annexin 2 localization and calcium metabolism in osteoclasts co-cultured with PCa cells, thus suggesting the capability of HO-1 to modify the bone tumor niche [36].

Considering the anti-tumoral role of HO-1 in PCa, by affecting diverse biological functions, and given its homeostatic activity, in this work we assessed the implications of HO-1 induction in the metabolic fate of PCa cells, analyzing different metabolic parameters such as glucose uptake, ATP production, oxygen consumption, LDH expression and activity and total mitochondrial mass after HO-1 pharmacological induction in PCa cells. Additionally, we performed a bioinformatics analysis to assess the clinical significance of *LDHA*, *LDHB* and *HMOX1* in PCa patients. Thus, we provide evidence on HO-1 involvement in the reprogramming of the metabolic status of PCa cells.

## 2. Materials and Methods

### 2.1. Cell Culture and Treatments

PC3 (CRL-1435, ATCC, USA) and C4-2B (CRL-3315, ATCC, USA) cells were routinely cultured in RPMI 1640 (GIBCO, Grand Island, NY, USA), supplemented with 100 U/mL of penicillin, 100 μg/mL of streptomycin, 0.5 μg/mL of amphotericin and 10% fetal bovine serum (FBS), at 37 °C in 5% CO_2_. MDA PCa 2b cells [37] were propagated in BRFF-HPC1 medium (AthenaES, Baltimore, MD, USA), supplemented with 100 U/mL of penicillin, 100 μg/mL of streptomycin and 20% FBS, at 37 °C in 5% CO_2_.

For pharmacological overexpression of HO-1, cells were cultured for 24 h in complete medium and were then incubated for 24 h in complete medium in the presence or absence of hemin (80 μM; Sigma-Aldrich, St. Louis, MO, USA).

### 2.2. Glucose Uptake

Glucose uptake rate was determined with the Glucose Uptake-Glo Assay (Promega, Madison, WI, USA), based on the detection of 2-deoxyglucose-6-phosphate (2DG6P), following manufacturer’s recommendations.

### 2.3. ATP Production

ATP levels were evaluated using the CellTiter-Glo Luminescent Cell Viability Assay (Promega, Madison, WI, USA), based in the mono-oxygenation of luciferin catalyzed by luciferase in the presence of Mg^2+^, ATP and O_2_, according to manufacturer’s guidelines.

### 2.4. Oxygen Consumption Rate and Extracellular Acidification Rate

The oxygen consumption rate (OCR) and the extracellular acidification rate (ECAR) were measured in cell cultures in DMEM without glucose, L-glutamine, phenol red, sodium pyruvate and sodium bicarbonate (Sigma-Aldrich, St. Louis, MO, USA) using the Seahorse XF Cell Mito Stress Test Kit and the Seahorse XFe96 Analyzer (Seahorse Bioscience, North Billerica, MA, USA) under basal conditions and in the presence of the inhibitor of the oxidative phosphorylation oligomycin (1 μM), the mitochondrial uncoupler carbonyl cyanide 4-(trifluoromethoxy)phenylhydrazone (FCCP; 0.25 μM for PC3 cells and 0.5 μM for C4-2B cells) and the inhibitors of complex III and I of the respiratory chain antimycin A (0.5 μM) and rotenone (0.5 μM). Basal respiration, ATP-linked respiration, proton leak, maximal respiratory capacity and non-mitochondrial respiration were calculated based on OCR.

### 2.5. Lactate Production

Extracellular lactate was determined in the conditioned media using the Lactate Colorimetric Assay Kit II (BioVision, Milpitas, CA, USA), following manufacturer’s instructions, and normalized to the number of cells.

### 2.6. Mitochondrial Membrane Potential

Mitochondrial integrity was estimated through analysis of the mitochondrial membrane potential. Cells were incubated with tetramethylrhodamine ethyl ester (TMRE; 100 nM; Invitrogen, Carlsbad, CA, USA) for 20 min at 37 °C, washed with phosphate buffered saline (PBS), trypsinized and resuspended with PBS. TMRE staining was then evaluated by flow cytometry (BD FACS Aria II cytometer, BD Biosciences, San Jose, CA, USA) and analyzed with FlowJo 7.6 software (Tree Star, Ashland, OR, USA). To determine the baseline, control cells were treated with the uncoupler FCCP (20 µM; Abcam, Cambridge, UK) for 10 min before the staining.

### 2.7. Total Mitochondrial Mass

After treatment, cells were incubated with MitoTracker Green FM (100 nM; cat. M7514, Invitrogen, Carlsbad, CA, USA) for 30 min at 37 °C, washed with PBS, trypsinized and resuspended with PBS. MitoTracker Green staining was then measured by flow cytometry (BD FACS Aria II cytometer, BD Biosciences, San Jose, CA, USA) and analyzed with FlowJo 7.6 software (Tree Star, Ashland, OR, USA).

### 2.8. RNA Isolation, cDNA Synthesis and RT-qPCR

Total RNA was extracted with Quick-Zol (Kalium Technologies, Bernal, Argentina) and reverse transcribed using the RevertAid RT kit (Fermentas, Waltham, MA, USA). Quantitative real-time PCR (qPCR) was performed on a QuantStudio 3 thermocycler (Applied Biosystems, Waltham, MA, USA) using FastStart Universal SYBR Green Master (ROX) 1X (Roche, Rotkreuz, Switzerland). Primer sequences (5′-3′) used were: *PPIA*: GGTATAAAAGGGGCGGGAGG and CTGCAAACAGCTCAAAGGAGAC; *PKM2*: TGCAGTGGAGCTCAGAGAGA and GTCTGAATGAAGGCAGTCCC; *ACO2*: ACAGCCTACTGGTGACTCGG and GCTCAAAGTGGCTCATCGC; *LDHA*: TTGTTGGGGTTGGTGCTGTTG and TGGTGTTCTAAGGAAAAGGCTG; *LDHB*: GGTTGAAAGTGCCTATGAAGTC and TACATGGAAGGCTCAGGAAGA; *SLC2A1*: GTCTGGCATCAACGCTGTCT; AACAGCGACACGACAGTGAA. Each sample was run in triplicate and *PPIA* was used as a reference gene. Data were obtained with the QuantStudio Design & Analysis Software v1.4.1 (Applied Biosystems, Waltham, MA, USA) and analysis was based on the ΔΔCt method [38].

### 2.9. Western Blot

Cells were lysed with RIPA buffer (Tris HCl 50 mM pH 7.4; NaCl 150 mM; EDTA 20 mM pH 8; sodium deoxycholate 1% *v*/*v*; SDS 0.1% *w*/*v*; Triton X-100 1% *v*/*v*, 1 mM Na_3_VO_4_, 20 mM NaF and 1 mM Na_4_P_2_O_7_, pH 7.9), incubated on ice for 20 min, centrifuged at 12,500 rpm for 20 min at 4 °C and the supernatant was collected. Protein concentration was determined using a bicinchoninic acid (BCA) protein assay kit (Sigma-Aldrich, St. Louis, MO, USA). Samples were then resolved on 10% SDS-PAGE and transferred to a nitrocellulose membrane (GE Healthcare, Little Chalfont, UK). Membranes were blocked with 5% dry non-fat milk in TBS containing 0.1% Tween-20 (TBS-T) for 40 min at room temperature and then incubated with specific primary antibodies overnight at 4 °C: anti-HO-1 (1:1000; cat. #5853, Cell Signaling, Danvers, MA, USA) and anti-β-actin (1:1000; cat. #3700S, Cell Signaling, Danvers, MA, USA). The next day, the membranes were incubated with anti-rabbit and anti-mouse secondary antibodies (1:5000; cat. #7074 and cat. #7076S, Cell Signaling, Danvers, MA, USA) for 1 h at room temperature. Protein bands were detected using EasySee Western Blot kit (TransGen Biotech, Beijing, China) and quantified by densitometry analysis using ImageJ software (NIH, Bethesda, MD, USA).

### 2.10. NAD(P)^+^ and NAD(P)H Measurement

Intracellular NAD(P)^+^ and NAD(P)H relative levels were determined using NAD/NADH-Glo Assay and NADP/NADPH-Glo Assay (Promega, Madison, WI, USA). For both couples, cells were lysed with base solution (Triton X-100 1% *v*/*v* in NaOH 0.2 N) and the lysate was split in two parts. To measure the oxidized forms (NAD^+^ and NADP^+^), samples were treated with HCl 0.4 N at 60 °C for 15 min and incubated at room temperature for 10 min, followed by the addition of Tris base 0.5 M and NAD/NADH or NADP/NADPH-Glo Detection Reagent. To measure the reduced forms (NADH and NADPH), samples were heated at 60 °C for 15 min and incubated at room temperature for 10 min, followed by the addition of HCl-Tris solution and NAD/NADH or NADP/NADPH-Glo Detection Reagent. The luminescent signal was recorded and NAD(P)^+^/NAD(P)H ratios and relative amounts were calculated.

### 2.11. Enzymatic Activity of Lactate Dehydrogenase (LDH)

After treatment, cells were incubated for 8 min with lysis solution (Triton X-100 9% *v*/*v* in PBS) and centrifuged at 200 g for 4 min. LDH activity was determined in the supernatant using the LDH-P UV AA Kit (technique with separate reagents at 30–37 °C; Wiener, Rosario, Argentina) and measuring absorbance at 340 nm. Values were normalized to total protein content.

### 2.12. Statistical Analysis

ATP Results are shown as mean ± standard deviation (SD) of at least three separate independent experiments unless stated otherwise. Data were analyzed using GraphPad Prism software (La Jolla, CA, USA) and Student’s *t* tests were used to ascertain statistical significance with a threshold of * *p* < 0.05, ** *p* < 0.01 and *** *p* < 0.001.

### 2.13. Bioinformatics Analysis

The public cancer database Oncomine (http://www.oncomine.org/; accessed on 8 June 2021) was searched to identify expression microarray datasets that compared gene expression in prostate adenocarcinoma versus normal prostate glands. As inclusion criteria, the datasets were required to be generated from human tumors and compare prostate adenocarcinoma versus normal prostate glands. Genes were ranked by their *p*-value for every dataset, scoring a gene rank. The median rank was defined as the median *p*-value rank across datasets, for each gene assessed. Alterations in gene expression were considered significant when presenting a *p* < 0.05 and having a fold change >1.5 and/or a gene rank within the top 10%. *LDHA* or *LDHB* were used as search terms, and the resulting studies were analyzed on the basis of healthy prostate gland versus prostate adenocarcinoma. Cited literature was reviewed to confirm that the analysis was as documented in the Oncomine database.

Expression of mRNA and clinical data for *LDHA* and *LDHB* from the Prostate Adenocarcinoma Project of The Cancer Genome Atlas (TCGA-PRAD) dataset (http://cancergenome.nih.gov/, accessed on 8 June 2021), which has RNAseq data from 497 prostate tumor samples and 52 non-tumoral adjacent tissue samples, were downloaded from the UCSC Xena platform [39] (accessed March 2021). Differential gene expression was calculated for primary tumor vs non-tumoral tissue or according to the International Society of Urologic Pathologists (ISUP) grading system [40]. Increasing or decreasing Jonckheere–Terpstra trend tests (with 500 permutations) were used to determine statistical trends between gene expression and ISUP grades.

To study the impact of expression levels on relapse-free survival (RFS) for PCa patients, one dataset was selected according to the following criteria: (1) the study included gene expression and clinical data for each patient with ≥5 years of follow-up; (2) the study consisted of ≥60 samples; (3) the study was published and available in public repositories. We used the dataset from Ross–Adams 2015 (GSE70770) [41], available in the Gene Expression Omnibus (GEO). It includes information on a prostate cancer patient cohort with 203 samples from men who had undergone radical prostatectomy and clinical follow-up of eight years, including relapse information (biochemical relapse). Biochemical relapse was defined according to the European Guidelines as a prostate specific antigen (PSA) persistent rise above 0.2 ng/mL). Tumor sample expression of 31,000 transcripts was measured by 47,000 probes using the Illumina HumanHT-12 V4.0 platform. Stata software (StataCorp LLC, College Station, TX, USA) was used to explore patient’s relapse free survival and to graph Kaplan–Meier curves. The cutoff expression value to stratify patients in high or low expression for each gene was established using the minimal *p*-value method from the Cutoff Finder Tool in R. To determine the effect on relapse-free survival for each gene, log-rank test and Cox proportional hazard model regression were used. Statistical significance was set as *p* < 0.05.

## 3. Results

### 3.1. HO-1 Impairs the Metabolic Status of PCa Cells

**Figure 1:**
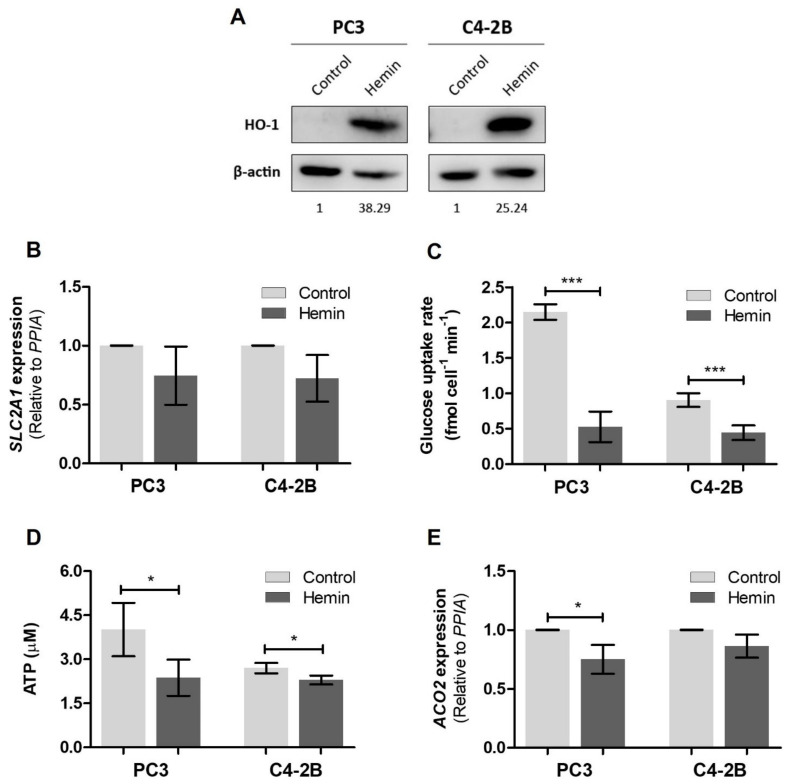
Effect of HO-1 pharmacological induction on the metabolism of PCa cells. (**A**) Western Blot analysis for HO-1 and β-actin in control and hemin treated (80 µM, 24 h) PC3 and C4-2B cells. Protein quantification, normalized to β-actin (loading reference) and to control lane, is indicated under each band. One representative of three independent experiments is shown. (**B**) *SLC2A1* (GLUT1) mRNA expression in PC3 and C4-2B cells under HO-1 induction assessed by RT-qPCR. Values were relativized using *PPIA* as the reference gene and normalized to the control. (**C**) Glucose uptake rate after hemin treatment in PC3 and C4-2B cell lines, determined using the Glucose Uptake-Glo Assay (Promega, USA). (**D**) ATP production in hemin treated PC3 and C4-2B cells, measured with the CellTiter-Glo Luminescent Cell Viability Assay (Promega, USA). (**E**) *ACO2* mRNA expression in PC3 and C4-2B cells under hemin treatment, assessed by RT-qPCR. Values were relativized using *PPIA* as reference gene and normalized to the control. (**B**–**E**): Data are presented as mean ± SD of three independent experiments, each performed in triplicate. Significant differences: * *p* < 0.05; *** *p* < 0.001.

To assess whether HO-1 induction impacts cellular energetics status, we performed metabolic assays in PCa cells after treatment with hemin (80 µM, 24 h), a specific inducer of HO-1 (Figure 1A and Figure S1). Since the glucose transporter GLUT1, encoded by the *SLC2A1* gene, shows higher expression levels in PCa [42], and its overexpression is a prognostic factor for PCa patients [43], we evaluated *SLC2A1* levels and glucose uptake under hemin treatment. Despite the fact that no significant variation was found in *SLC2A1* expression (Figure 1B), there was a significant reduction in the glucose consumption rate in both androgen receptor (AR) expressing cells (C4-2B) and AR non-expressing cells (PC3) after HO-1 induction (*p* < 0.001) (Figure 1C). Consistently, the intracellular ATP concentration was significantly reduced in these cell lines after HO-1 induction (*p* < 0.05) (Figure 1D). Further, we assessed the levels of *ACO2*, which has a tumor-promoting role in PCa, [44] and found a significantly decreased expression only in PC3 cells (*p* < 0.05), suggesting a partial blockade of the TCA cycle under HO-1 induction (Figure 1E).

**Figure 2:**
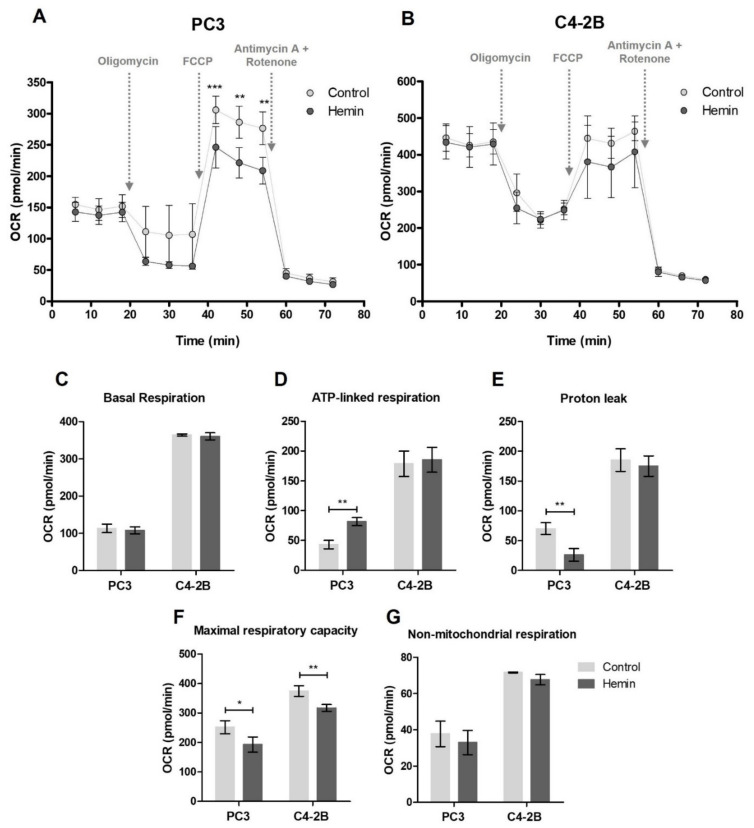
Oxygen consumption in PCa cells after HO-1 induction. (**A**) PC3 and (**B**) C4-2B oxygen consumption rate (OCR) after hemin treatment, determined in basal conditions and in response to 1 μM oligomycin, 0.25 μM FCCP for PC3 cells or 0.5 μM FCCP for C4-2B cells and 0.5 μM antimycin A + 0.5 μM rotenone using the Seahorse XF Cell Mito Stress Test Kit (Seahorse Bioscience, USA). Bar graphs showing the quantification of: (**C**) Basal respiration, (**D**) ATP-linked respiration, (**E**) proton leak, (**F**) maximal respiratory capacity and (**G**) non-mitochondrial respiration. Data are presented as mean ± SD of six replicates. Significant differences: * *p* < 0.05; ** *p* < 0.01; *** *p* < 0.001.

We next examined whether these findings could be associated with an impairment in the mitochondrial function, evaluating the oxygen consumption. Particularly, in the presence of the uncoupler FCCP, the oxygen consumption rate (OCR) was significantly reduced in PC3-treated cells (*p* < 0.01) (Figure 2A), evidencing a lower maximal respiratory capacity and, as a consequence, a smaller reservoir capacity, while no alteration was detected in C4-2B cells (Figure 2B). Interestingly, C4-2B cells presented similar OCR levels both in basal conditions and after the treatment with FCCP, independently of HO-1 induction. Further, basal respiration, ATP-linked respiration, proton leak, maximal respiratory capacity and non-mitochondrial respiration were calculated (Figure 2C–G). It is worth noting the decrease in the maximal respiratory capacity detected in both cell lines after hemin treatment (*p* < 0.05 for PC3 cells and *p* < 0.01 for C4-2B cells) (Figure 2F).

**Figure 3:**
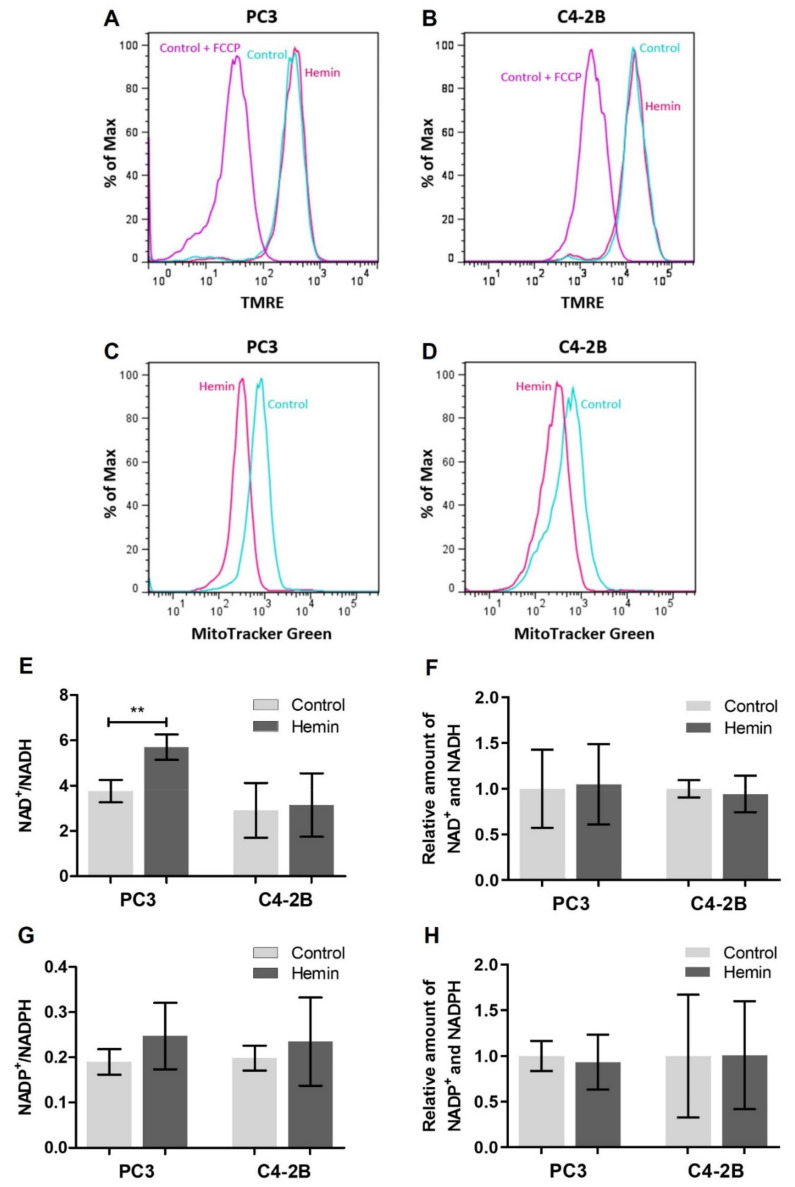
Mitochondrial status and redox balance in PCa cell lines under HO-1 induction. (**A**) PC3 and (**B**) C4-2B mitochondrial integrity evaluated after hemin treatment by flow cytometry using the potential-sensitive dye TMRE. Pre-treatment of control cells with the potent uncoupler of mitochondrial oxidative phosphorylation, FCCP (carbonyl cyanide 4-(trifluoromethoxy)phenylhydrazone), was used to determine the baseline. One representative of three independent experiments is shown. (**C**) PC3 and (**D**) C4-2B total mitochondrial mass was assessed after hemin treatment by flow cytometry using MitoTracker Green. One representative of three independent experiments is shown. (**E**) NAD^+^/NADH ratio and (**F**) total amount of NAD^+^ and NADH (normalized to the control) were determined using the NAD/NADH-Glo Assay (Promega, USA). Data are presented as mean ± SD of three independent experiments, each performed in triplicate. (**G**) NADP^+^/NADPH ratio and (**H**) total amount of NADP^+^ and NADPH (normalized to the control) were measured with the NADP/NADPH-Glo Assay (Promega, USA). Results are shown as mean ± SD of three independent experiments, each performed in triplicate. Significant differences: ** *p* < 0.01.

Moreover, in order to study the effect of HO-1 pharmacological overexpression on the mitochondrial integrity and mass, we performed flow cytometry assays with fluorescent dyes. First, we used the cationic potential-sensitive dye TMRE, which only accumulates in intact mitochondria, and the signal was not modified in response to hemin treatment in either of the cell lines assessed (Figure 3A,B). Thus, this result suggested that the mitochondrial integrity was not affected in our experimental conditions. We also used MitoTracker Green, which labels mitochondria in an independent membrane potential manner, therefore providing a readout relating purely to the mitochondrial mass. Results showed that mitochondrial mass was reduced under HO-1 induction in both cell lines (Figure 3C,D). Together, these results indicate that after hemin treatment the number of damaged mitochondria is reduced, which could be reflecting the homeostatic potential of HO-1.

Considering that redox imbalance is involved in cancer progression altering gene expression, protein stability and cellular programs [45,46,47], we measured redox couples NAD^+^/NADH and NADP^+^/NADPH in PCa cells under hemin treatment. A significant increase in the ratio NAD^+^/NADH was detected in hemin treated PC3 cells (*p* < 0.01) without changes in the total amount of both species (Figure 3E,F). These results might be related to the TCA cycle being partially blocked and a consequent weakened NADH recycling. No significant alterations were observed either in the couple NADP^+^/NADPH in PC3 cells (Figure 3G,H) or in both coenzyme couples in C4-2B cells in the experimental conditions assayed (Figure 3E–H).

**Figure 4:**
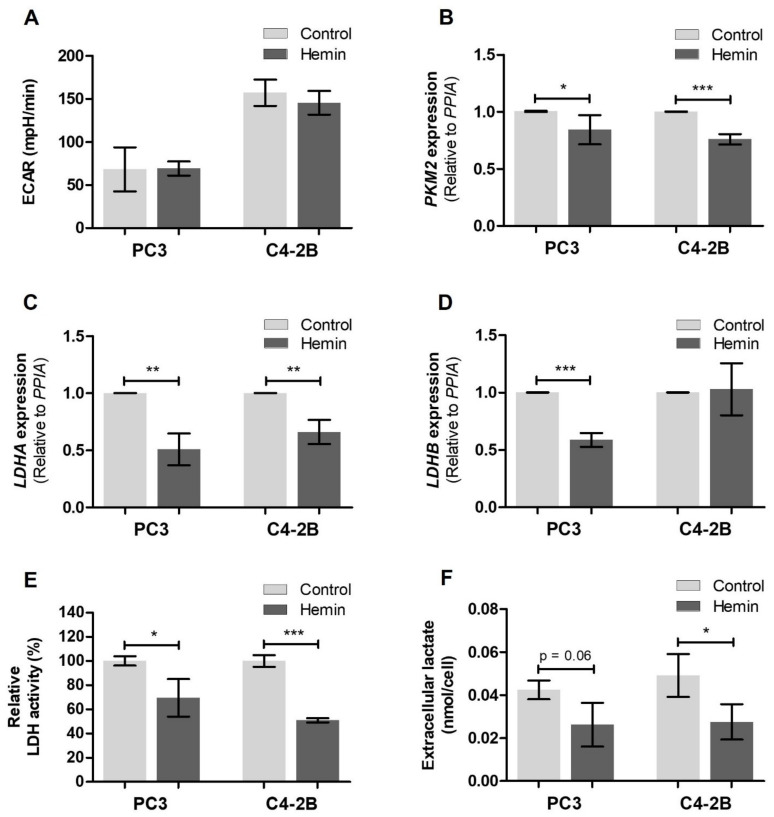
Modulation of aerobic glycolysis in PCa cells after HO-1 induction. (**A**) Extracellular acidification rate (ECAR) measured in response to 1 μM oligomycin in control and hemin treated PC3 and C4-2B cells using the Seahorse XF Cell Mito Stress Test Kit (Seahorse Bioscience, USA). Results are shown as mean ± SD of six replicates. (**B**) *PKM2*, (**C**) *LDHA* and (**D**) *LDHB* mRNA expression in PC3 and C4-2B cells under HO-1 induction was assessed by RT-qPCR. Values were relativized using *PPIA* as reference gene and normalized to control. Data are presented as mean ± SD of three independent experiments, each performed in triplicate. (**E**) LDH activity determined in PC3 and C4-2B hemin treated cells using the LDH-P UV AA Kit (Wiener, Argentina). Values were relativized to total protein content and normalized to control. Results are shown as mean ± SD of three independent experiments. (**F**) Extracellular lactate levels were measured in the conditioned media of hemin treated PC3 and C4-2B cells using the Lactate Colorimetric Assay Kit II (BioVision, USA) and were normalized to the number of cells. Data are presented as mean ± SD of three independent experiments, each performed in duplicate. Significant differences: * *p* < 0.05; ** *p* < 0.01; *** *p* < 0.001.

Given that aerobic glycolysis and lactate production have a preponderant role in cancer progression, we sought to assess some parameters of this metabolic pathway. We first evaluated the glycolytic function through the determination of the extracellular acidification rate (ECAR). No differences were found in ECAR after hemin treatment either in PC3 or C4-2B cells, compared with their respective controls (Figure 4A). However, we found a significant reduction in the transcriptional expression of pyruvate kinase M2 (PKM2) (*p* < 0.05 for PC3 cells and *p* < 0.001 for C4-2B cells) (Figure 4B), a key glycolytic enzyme that regulates the final rate-limiting step, catalyzing the conversion of phosphoenolpyruvate (PEP) to pyruvate [48]. In addition, we studied the mRNA levels of the main LDH isoforms and detected reduced *LDHA* expression in PC3 and C4-2B cells under hemin treatment (*p* < 0.01) (Figure 4C), while *LDHB* expression was significantly lower in treated PC3 (*p* < 0.001) and no changes were detected in C4-2B cells (Figure 4D). Concomitantly, we observed a lower LDH activity in both cell lines (*p* < 0.05 for PC3 cells and *p* < 0.001 for C4-2B cells) (Figure 4E), as well as a decreased amount of lactate in the conditioned media, which was marginally significant in PC3 cells (*p* = 0.06) and statistically significant in C4-2B cells (*p* < 0.05) (Figure 4F). Furthermore, similar results in glucose uptake, mitochondrial integrity and extracellular lactate levels were obtained using another AR sensitive PCa cell line, MDA PCa 2b (Figure S2).

Altogether, our findings demonstrate a shift in the metabolic profile of PCa cells under HO-1 induction, slowing down the aerobic glycolytic pathway and maintaining cellular homeostasis, suggesting this could be one of the mechanisms through which HO-1 exerts its anti-tumoral role.

### 3.2. Clinical Relevance of LDHA, LDHB and HMOX1 in PCa

Considering the importance of LDH in cancer metabolism and HO-1-mediated effects on this enzyme transcription and activity in PCa cells, we searched public database repositories and performed a bioinformatics analysis to determine the clinical significance of *LDHA*, *LDHB* and *HMOX1*.

**Figure 5:**
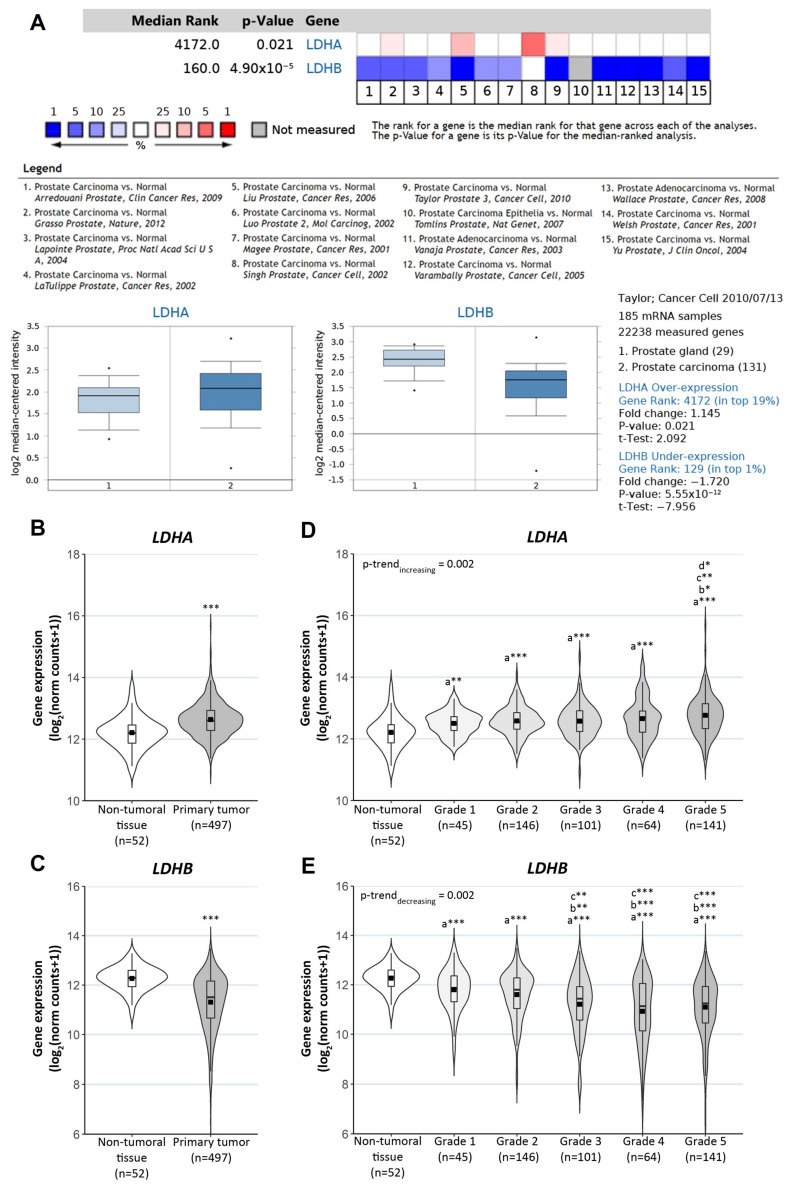
Clinical relevance of *LDHA* and *LDHB* expression in PCa. (**A**) Meta-analysis of microarray datasets on the public cancer database Oncomine for *LDHA* and *LDHB* expression (*n* = 973 patient samples). One representative box plot is shown for each gene comparing its expression profile between group 1: Prostate Gland and group 2: Prostate Adenocarcinoma. Analysis of differential gene expression using RNAseq data from TCGA-PRAD: (**B**) *LDHA* and (**C**) *LDHB* mRNA levels in primary tumor samples (*n* = 497) and non-tumoral adjacent tissue samples (*n* = 52). (**D**) *LDHA* and (**E**) *LDHB* expression levels in PCa samples categorized according to the ISUP grading system. Significant differences: * *p* < 0.05; ** *p* < 0.01; *** *p* < 0.001; the letter represents which group sample was considered as a reference: a = non-tumoral tissue, b = grade 1, c = grade 2 and d = grade 3; *p*-trend values correspond to Jonckheere-Terpstra trend test.

First, we examined the public cancer database Oncomine. The meta-analysis of multiple microarray datasets showed a significantly lower expression of *LDHB* in prostate adenocarcinomas compared with normal prostate glands (Median Rank: 160; *p* = 4.9 × 10^−5^; *n* = 973 patient samples), while *LDHA* expression levels presented no significant differences (Figure 5A).

In addition, we analyzed the Prostate Adenocarcinoma Project of The Cancer Genome Atlas (TCGA-PRAD) RNAseq dataset. We detected increased *LDHA* expression (*p* < 0.001) and reduced *LDHB* expression (*p* < 0.001) in tumor samples compared with non-tumoral adjacent tissue samples (Figure 5B,C), suggesting that lactate production would be favored. Further, we observed that, as the ISUP grade increased, there was a statistical significant trend of higher *LDHA* and lower *LDHB* expression (*p*-trend = 0.002 for both comparisons) (Figure 5D–E).

**Figure 6:**
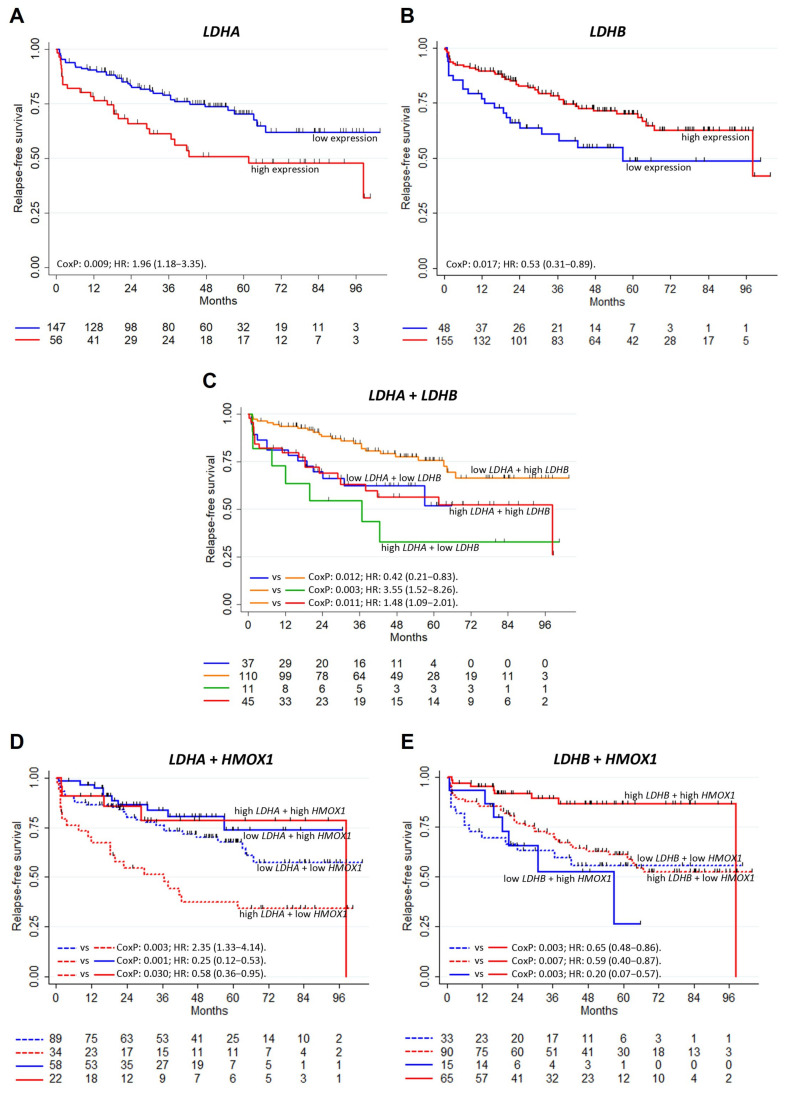
Analysis of *LDHA*, *LDHB* and *HMOX1* as risk predictors of clinical outcome in PCa. Relapse-free survival (RFS) for PCa patients from Ross–Adams dataset (GSE70770)^39^ (*n* = 203). Kaplan–Meier curves for RFS of PCa patients with low and high expression levels of (**A**) *LDHA* and (**B**) *LDHB,* individually; (**C**) *LDHA* and *LDHB*, (**D**) *LDHA* and *HMOX1* and (**E**) *LDHB* and *HMOX1* combined. Cox P and HR: hazard ratio (95% confidence interval) are specified. Vertical marks show censored patients. Statistical significance was set at *p* < 0.05.

Finally, to assess whether *LDHA* and *LDHB* expression were related to the clinical outcome of PCa, we used the Ross–Adams dataset (GSE70770) [41] to evaluate the relapse-free survival (RFS) of PCa patients that had undergone radical prostatectomy. Results showed that higher expression of *LDHA* was associated with a worse RFS, while the opposite was observed for *LDHB* (HR: 1.96, *p* = 0.009 for *LDHA*; HR: 0.53, *p* = 0.017 for *LDHB*) (Figure 6A,B). When both variables were analyzed together (Figure 6C), we observed a subpopulation with combined low *LDHA*/high *LDHB* that displayed an improved outcome compared with those that had low *LDHA*/low *LDHB* (*p* = 0.012), high *LDHA*/low *LDHB* (*p* = 0.003), and high *LDHA*/high *LDHB* (*p* = 0.011).

Regarding *HMOX1*, we previously performed a similar analysis using these three databases, which revealed no significant differences in mRNA expression levels among tumoral and non-tumoral prostate tissue, but demonstrated that PCa patients with higher expression of *HMOX1* showed longer RFS (HR: 0.5, *p* = 0.021) [36]. Herein, we found that the association between higher *HMOX1* levels and longer RFS was not modified by LDHA expression (Figure 6D). On the other hand, longer RFS was observed in patients with high *LDHB* only when they also exhibited high *HMOX1* (Figure 6E; *p* = 0.007). These results suggest that the worst RFS resulted for patients with low *HMOX1* and high *LDHA* expression, and the best prognosis, for those with high *HMOX1* and high *LDHB* expression.

## 4. Discussion

In PCa, as in other malignancies, the reprogramming of metabolic pathways is pivotal for sustaining growth and proliferation. In this work, we are reporting the impact of HO-1 pharmacological induction in halting the exacerbated glycolytic metabolism of PCa cells. Furthermore, we have discovered that a PCa patient profile with increased HO-1 and LDHB mRNA levels has the best prognosis in terms of increased RFS.

Deregulating cellular energetics has emerged as a hallmark for the maintenance of tumor cells high proliferative rate [4]. The importance of cancer metabolic shift originally proposed by Warburg [5,6] was later evidenced by the various connections among the different signaling pathways that include oncoproteins, tumor suppressor proteins and key enzymes involved in the energetic metabolism [49]. Thus, alterations in the expression of a single gene, as a consequence of several signaling cascades, might be amplified in different metabolic processes [50]. Further, variations in the expression of a low number of genes can drive changes in metabolic pathways, resulting in an adaptive advantage for cancer cells. The signaling network cross-talk characteristic of the advanced prostatic tumors shows that targeting one pathway might not be sufficient and appears to be necessary to decode the intricacy of the metabolic reprogramming in order to design new therapies.

Although HO-1 is normally found at low levels, exerting vital metabolic functions that maintain cellular homeostasis, its expression is highly upregulated under oxidative stress and inflammation, having a protective pleiotropic role [51]. We have previously demonstrated that, beyond its homeostatic function, HO-1 has an anti-tumoral role in PCa, negatively modulating angiogenesis and slowing down tumoral growth in vivo, as well as decreasing cellular proliferation, migration and invasion in vitro [29]. We have also found that HO-1 overexpression downregulates the expression of pro-inflammatory and pro-angiogenic genes, represses the NF-κB pathway and inhibits STAT3 signaling, disrupting its association with AR and, therefore, the transcriptional activity of this receptor [29,30,31]. Considering that HO-1 partially localizes in the nucleus in PCa human samples, and partially translocates to the nucleus under induction in different cell types [52,53], it might need to partner up with other transcription factors or co-regulators of transcription to exert its regulatory function, given that it does not contain DNA binding motifs. Moreover, it has been recently reported that nuclear HO-1 might influence gene transcription by regulating the stability of DNA G-quadruplexes structures [54].

In line with our findings, HO-1 anti-tumoral functions in breast cancer [55], hepatocellular carcinoma [56] and colorectal cancer have also been reported [57,58]. However, there are studies showing that HO-1 can play an opposite pro-tumoral effect in head and neck squamous cell carcinoma [59], non-small cell lung cancer [60] and gliomas [61]. This HO-1 dual role in cancer, which seems to be context, tumor microenvironment and cell type-dependent [62], could be partially explained by HO-1 subcellular localization [63] and its dependence on tissue conditions to act as a pro- or anti-angiogenic factor [64,65].

HO-1 properties are partially attributed to its enzymatic products (i.e., carbon monoxide, biliverdin/bilirubin) and have been shown to be protective in some metabolic diseases and pathophysiologic conditions, including atherosclerosis, diabetes, ischemia and cancer [24,25,66,67,68,69]. A recent study by Lilljebjörn et al., which evaluated HO-1 expression in mice tissues upon *E. coli* infection, showed that the increase of HO-1 itself and/or carbon monoxide are capable of preventing the development of prostatic intraepithelial neoplasias and the growth of prostate tumors, apparently through the modulation of pathways involved in fatty acid and lipid metabolism in macrophages and epithelial cells [70]. Moreover, mitochondrial HO-1 expression modulates O_2_ uptake and ROS production in the liver [71], and provides cytoprotection against mitochondria-mediated cell death in lung epithelial cells [72].

Herein, we demonstrated that HO-1 pharmacological induction significantly altered key parameters of cellular metabolism in PCa cell lines. Despite not having observed changes in the ECAR, we were able to verify a decrease in glucose consumption and ATP production in both cell lines, indicating a reduction in the metabolic rate under HO-1 overexpression. We found that the OCR was significantly decreased in PC3; on the other hand, no alteration was observed for C4-2B. The reduced maximal respiratory capacity in PC3 cells could be associated with the lower total mitochondrial mass, while the diminished oxygen consumption linked to the proton leak might be related to an enrichment of the functional mitochondrial pool. Interestingly, C4-2B cells might be at their maximum respiratory capacity, as suggested by the similar basal respiration and maximal respiratory levels observed in the OCR assay.

Massie et al. reported that AR participates in PCa energy reprogramming, modifying the expression of multiple mediators in various catabolic and anabolic processes [73]. This report shows that androgen-stimulated PCa cells have higher glucose consumption and lactate production, while OCR is not modified. These data support our findings in the AR-dependent cell line C4-2B and our previous report demonstrating that hemin treatment impairs AR signaling in LNCaP cells [31]. On the other hand, the brake on energy metabolism imposed by the presence of hemin would justify reports that show the inhibition of cell proliferation and the delay in the growth of PC3-derived tumors with HO-1 over-expression [29].

It is well known that p53 regulates glycolysis and oxidative phosphorylation [74]. Consequently, p53 deficiency tends to decrease the efficiency of mitochondrial respiration, favoring glycolysis. Thus, our results suggest that hemin treatment favors the reprogramming of PC3 cells (p53 null) by restricting aerobic glycolysis. Similarly, other authors documented that the induction of HO-1 or treatment with CO reduces the methylation of PFK2 (phosphofructokinase 2), preventing the activation of the limiting enzyme of glycolysis PFK1 (phosphofructokinase-1), and thus deriving the use from glucose to the pentose phosphate pathway (PPP) [75]. Accordingly, in tumor cells, PKM2 also favors PPP since it has a lower efficiency than the M1 isoform in the conversion of phosphoenolpyruvate to pyruvate during glycolysis [7]. In this work, we were able to detect a significant decrease in *PKM2* expression in both PCa cell lines, reflecting again the impairment of the glycolytic pathway under hemin treatment.

The changes evidenced at the level of glycolysis and the expression of genes involved in energy metabolism could be indicators of alterations in the amount or integrity of mitochondria. However, when HO-1 was induced in PCa cells, no modifications were seen in the membrane potential, but the mitochondrial mass decreased. Thus, these results suggest that, after hemin treatment, the number of damaged mitochondria was reduced, reflecting the homeostatic potential of HO-1. Previously, we demonstrated that HO-1 contributes to the preservation of a healthy mitochondrial population in astroglial cells challenged with Mn^2+^ [76]. Similarly, Hull et al. showed that HO-1 overexpression exerted cellular protection through the maintenance of the mitophagic pathway at homeostatic levels in mice hearts [77].

HO-1 is implicated in glucose catabolism and the production of NADH [25]. When measuring the NAD^+^/NADH ratio in PC3 cells, a significant increase mediated by HO-1 induction was observed. In line with our results, Santidrian et al. documented that breast cancer tumor growth and metastasis were inhibited when complex I activity was enhanced and the resulting NAD^+^/NADH ratio increased [78].

Further, our studies also unveiled that hemin treatment impacts on LDH isoforms transcriptional expression and downregulates LDH activity with the concomitant decrease of extracellular lactate concentration, thus supporting our hypothesis of HO-1 as a key player modulating the energetic metabolism of PCa.

Moreover, we were specifically interested in the association of *LDHA*, *LDHB* and *HMOX1* in the prognosis of PCa. Bioinformatics analysis revealed that high *LDHA* or low *LDHB* expression was associated with worse RFS. We previously demonstrated that patients with low *HMOX1* expression had poorer RFS [36]. When combining the expression levels of *HMOX1* and *LDHA*, we showed that the worst risk was observed for patients with low *HMOX1* and high *LDHA*. We did not find differences in RFS for the other groups. Similarly, we found that patients with high *HMOX1* and high *LDHB* had better RFS compared to the other groups. In line with our findings, two additional reports evaluated the association between LDH blood concentration at diagnosis and PCa death, and both reported high LDH as a predictor of worse outcomes [79,80].

Altogether, our results demonstrate that HO-1 is implicated in the reprogramming of the metabolic status of PCa cells, leading to a less aggressive phenotype of the disease.

## 5. Conclusions

In vitro experiments showed HO-1-mediated effects on the cellular metabolism of PCa cells after HO-1 induction with hemin, including alterations to both LDH transcription and activity, which resulted in a slowdown of the aerobic glycolytic pathway. Furthermore, bioinformatics analysis demonstrated that increasing ISUP grades are associated with higher *LDHA* and lower *LDHB* expression, and that PCa patients with low *HMOX1* and high *LDHA* expression presented the worst prognosis in terms of RFS, while those with high *HMOX1* and high *LDHB* expression had the best outcome. Finally, as HO-1 specific inductor hemin is FDA approved, this study reaffirms HO-1 pharmacological induction as a promising potential therapy for PCa tumors.

## Data Availability

The publicly available datasets analyzed in this study can be found here: Oncomine database (http://www.oncomine.org; accessed on 8 June 2021), Cancer Genome Atlas dataset (http://cancergenome.nih.gov/; accessed on 8 June 2021 using the UCSC Xena platform [39]), Gene Expression Omnibus (GSE70770).

## Supplementary Materials

The following are available online at https://www.mdpi.com/article/10.3390/antiox10060966/s1, Figure S1: HO-1 induction by hemin treatment in PCa cells; Figure S2: Impact of HO-1 induction in MDA PCa 2b cells.
