# Supplementary material for: HO-1 Modulates Aerobic Glycolysis through LDH in Prostate Cancer Cells
Source: Antioxidants (Basel). 2021 Jun 16;10(6):966. doi: 10.3390/antiox10060966 (PMC8235201; doi:10.3390/antiox10060966)
Supplement: Supplementary file 1 [file antioxidants-10-00966-s001.zip › antioxidants-1245875-SI.pdf]

antioxidants MDPI

Supplementary Materials:

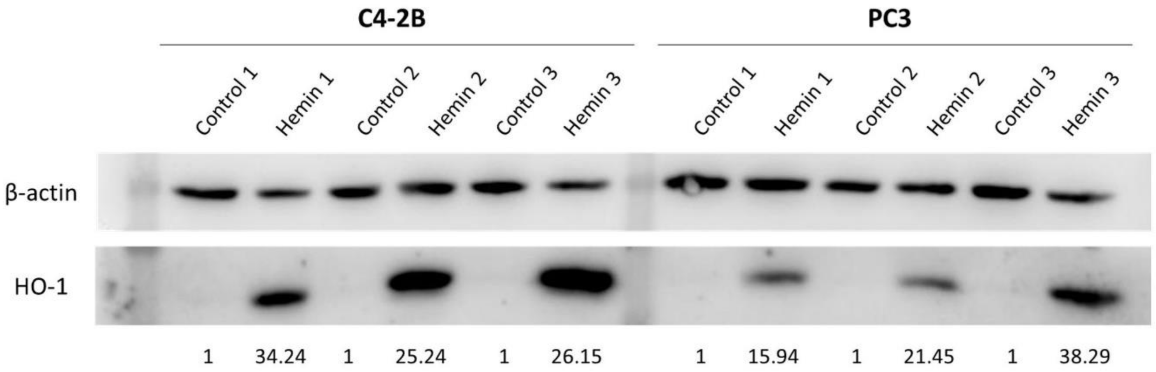

Figure S1. HO-1 induction by hemin treatment in PCa cells. Western Blot analysis for HO-1 and β-actin in control and hemin treated (80 μM, 24 h) PC3 and C4-2B cells. Protein quantification, normalized to β-actin (loading reference) and to control lane, is indicated under each band. Three independent experiments are shown.

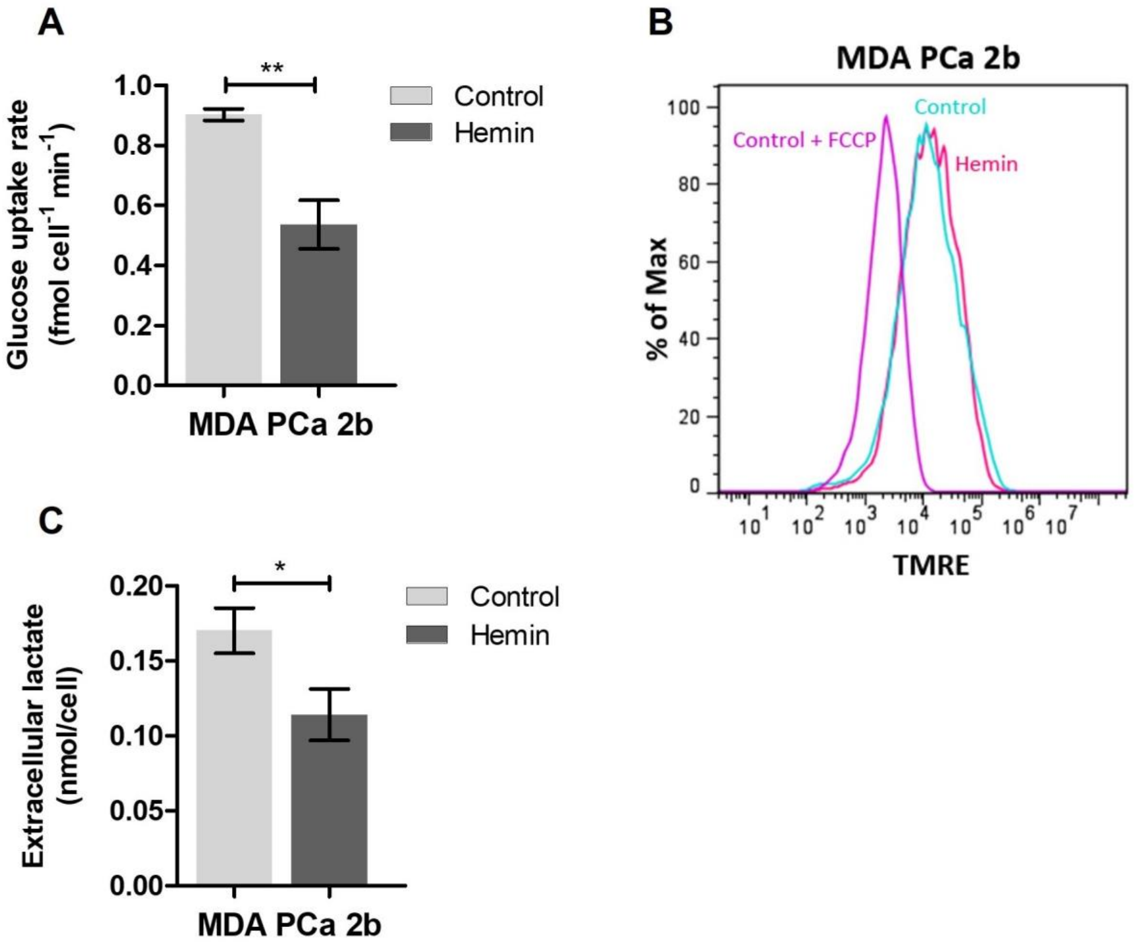

Figure S2. Impact of HO-1 induction in MDA PCa 2b cells. (A) Glucose uptake rate after hemin treatment (80 μM, 24 h), was determined using the Glucose Uptake-Glo Assay (Promega, USA). **(B)** Mitochondrial integrity was evaluated after hemin treatment by flow cytometry using the potential-sensitive dye TMRE. Pre-treatment of control cells with the uncoupler FCCP was used to determine the baseline. One representative of three independent experiments is shown. **(C)** Extracellular lactate levels measured in the conditioned media of hemin treated cells using the Lactate Colorimetric Assay Kit II (BioVision, USA) and normalized to the number of cells. Significant differences: * $p < 0.05$; ** $p < 0.01$.
